# *CCL24/CCR3* axis plays a central role in angiotensin II–induced heart failure by stimulating M2 macrophage polarization and fibroblast activation

**DOI:** 10.1007/s10565-022-09767-5

**Published:** 2022-09-22

**Authors:** Zhen Wang, Hongfei Xu, Miao Chen, Yunlong Lu, Liangrong Zheng, Liang Ma

**Affiliations:** 1 Department of Cardiothoracic Surgery, the First Affiliated Hospital, School of Medicine, Zhejiang University, Hangzhou, 310003 Zhejiang China; 2 Department of Cardiology, the First Affiliated Hospital, School of Medicine, Zhejiang University, Hangzhou, 310003 Zhejiang China

**Keywords:** Heart failure, *CCL24*, M2 macrophage, Cardiac fibroblast

## Abstract

**Aims:**

We aimed to investigate the effect and mechanism of pleiotropic *chemokine CCL24* in heart failure.

**Methods and results:**

Compared with normal donators, the expression of *CCL24* and number of cardiac M2 macrophages in heart were higher in heart failure patients, the same as plasma *CCL24*. Treatment with *CCL24 antibody* hindered Ang II (1500 ng/kg/min)–induced cardiac adverse remodeling through preventing cardiac hypertrophy and fibrosis. RNA-seq showed that *CCL24/CCR3* axis was involved in immune and inflammatory responses. Single-cell analysis of cytometry by time of flight (CyTOF) revealed that CCL24 antibody decreased the M2 macrophage and monocyte polarization during Ang II stimulation. Immunofluorescence co-localization analysis confirmed the expression of *CCR3* in macrophage and fibroblasts. Then, in vitro experiments confirmed that *CCL24*/*CCR3* axis was also involved in cardiac primary fibroblast activation through its G protein–coupled receptor function.

**Conclusion:**

*CCL24/CCR3* axis plays a crucial part in cardiac remodeling by stimulating M2 macrophage polarization and cardiac fibroblast activation.

**Graphical abstract:**

Cardiac M2 macrophages, CCL24 and circulation CCL24 increased in heart failure patients. Treatment with CCL24 Ab hindered Ang II induced cardiac structural dysfunction and electrical remodeling. In CCL24 Ab group RNA-seq found that it was related to immune responses and hypertrophic cardiomyopathy, CytoF revealed M2 macrophages and monocytes d**e**creased obviously. In vitro,CCL24 promoted activation and migration of cardiac fibroblast.

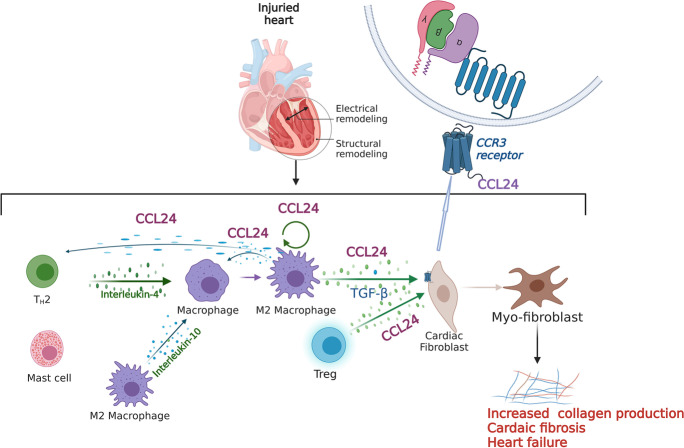

**Supplementary Information:**

The online version contains supplementary material available at 10.1007/s10565-022-09767-5.

## Introduction

Heart failure (HF) is a crucial health problem with massive morbidity and mortality (Murphy et al. 2020), which is the end-stage state of various heart diseases characterized by the imbalance of ECM (extracellular matrix) production and degradation, the decreased pumping capacity of the heart, and the disorder of cardiac electrical conduction (Curley et al. 2018). It is urgent to reveal the cause of continuous progression in heart failure and to find an effective therapeutic method of it.

Recently, the CANTOS trial, which targeted IL-1β with the monoclonal antibody, contributed to improved heart failure outcomes in myocardial infarction patients, raises the renewed interest in inflammation of heart failure (Shetelig et al. 2018; Dong et al. 2012; Damås et al. 2000a; Frangogiannis 2014) Chemokine is a family of inflammatory cytokines which are able to cause directional migration and activation of immune cells (Caidahl et al. 2019; Noels et al. 2019; Frangogiannis and Entman 2004; Liehn et al. 2011). Congestive heart failure (CHF) patients have significantly elevated levels of several chemokines, which may imply previously unrecognized pathogenic factors in CHF (Damås et al. 2000b; Aukrust et al. 1998).

*CCL24*, one of the pleiotropic chemokines, was shown to be involved in inflammatory reactions through its receptor *CCR3*, which specifically participates in macrophages and T helper cell differentiation (Sallusto et al. 1997; Ochi et al. 1999). Recently, *CCL24* has exhibited pro-fibrotic and pro-inflammatory effects in the lungs, skins, and livers (Segal-Salto et al. 2020; Mor et al. 2019; Kohan et al. 2010; Foster et al. 2015). In addition, *CCL24* was shown to be involved in cardiomyocyte proliferation, and it was one of the inflammation markers of the cardiac aging characteristic, and was positively associated with the recession in early-to-late LV filling ratio of the aging heart (Ma et al. 2015; Pinto et al. 2014). Moreover, CCL24 expression was significantly reduced in the treatment of TAC-induced cardiac hypertrophy and heart failure with sodium-glucose co-transporter 2 inhibitors (TD139). Therefore, we speculated that *CCL24/CCR3* is involved in cardiac remodeling during heart failure, and we planned to investigate the role and mechanism of the *CCL24/CCR3* axis in Ang II–induced heart failure.

We revealed that the expression of cardiac *CCL24* and plasma *CCL24* were higher in heart failure patients compared with normal donators, and blocking *CCL24* in mice alleviated Ang II–induced electrical remodeling and heart failure. In mechanisms, *CCL24/CCR3* axis participated in cardiac remodeling by stimulating M2 macrophage polarization. And in vitro, we found that *CCL24/CCR3* was also involved in primary cardiac fibroblast activation. In light of these results, we provided an effective therapeutic method for heart failure and cardiac remodeling.

## Results

### Increased levels of cardiac M2 macrophages, cardiac CCL24 expression, and circulation CCL24 in heart failure patients

**Fig. 1:**
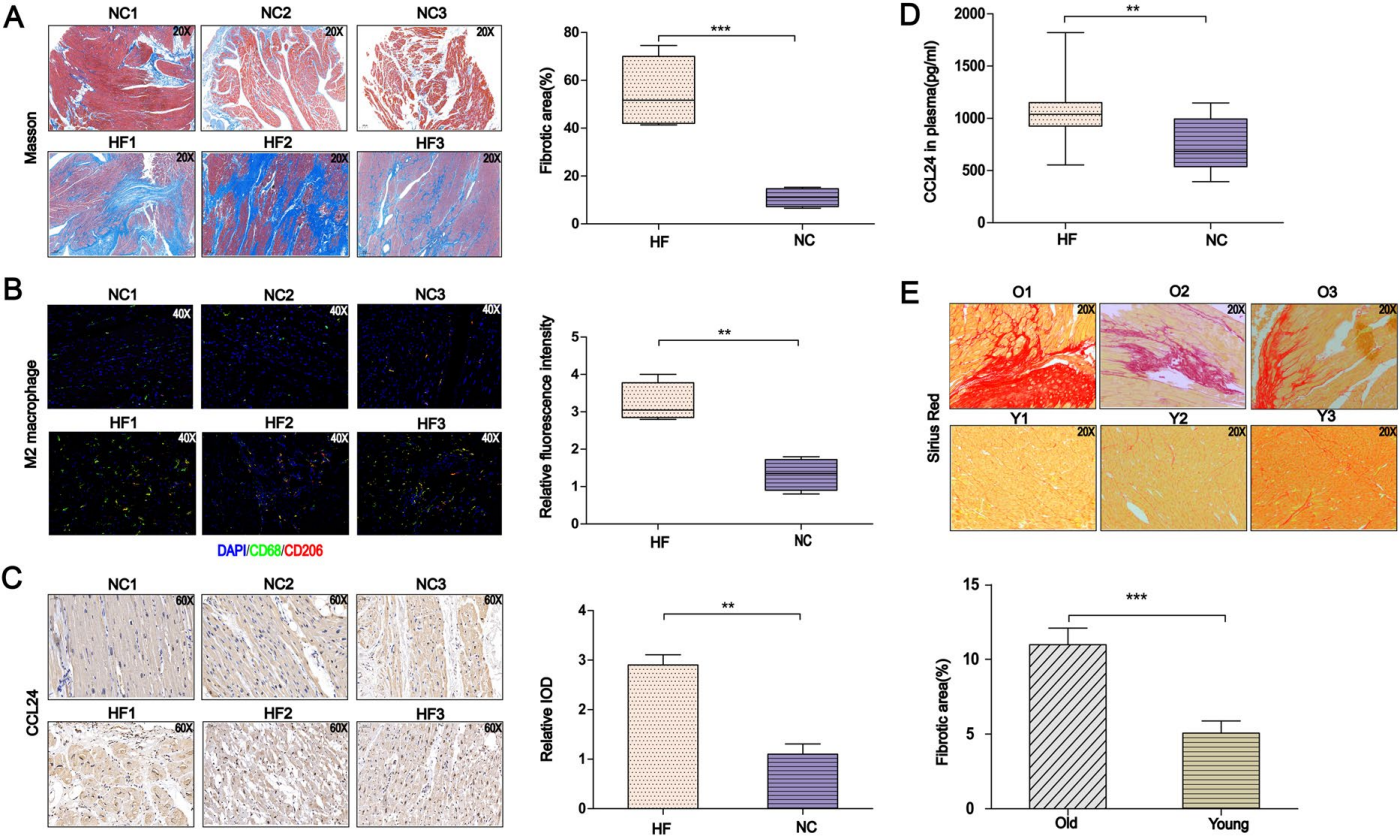
Correlation between CCL24/CCR3 axis and heart failure. **A** Masson’s trichrome staining of myocardial fibrosis in heart failure patient and donor cardiac tissue, scale bar in panel **B** is 200 μm. The data are represented as the means ± SEM (*n* = 5; unpaired Student’s *t* test; *P* < 0.0001: normal patient group vs. heart failure patient group). **B** Immunofluorescence staining of M2 macrophage in the heart tissue of heart failure and donor patients, scale bar in panel **A** is 500 μm. **C** Immunohistochemical staining of CCL24 in the heart tissue of heart failure and donor patients, scale bar in panel **A** is 500 μm. **D** The level of CCL24 in the plasma of heart failure patient and healthy donor. The data are represented as the means ± SEM (*n* = 14; unpaired Student’s *t* test; *P* < 0.05). **E** Sirius red staining of myocardial fibrosis in old (up) and young (down) mice, scale bar in panel **D** is 50 μm. The data are represented as the means ± SEM (*n* = 3; unpaired Student’s *t* test; *P* = 0.0014)

Firstly, the cardiac fibrosis in heart failure patients was significantly worse than in healthy control subjects (Fig. 1A). Because *CCL24* is mainly secreted by M2 macrophages, we found that heart failure patients had significantly elevated levels of M2 macrophages and cardiac *CCL24* expression compared with healthy donor individuals (Fig. 1B–C). Then, we revealed that the level of plasma *CCL24* in heart failure is higher than healthy volunteers (Fig. 1D). According to a previous study, the level of *CCL24* in old mice’s cardiac tissues was higher than in young mice; then, we found that the myocardial fibrosis in old mice’s hearts was even worse than in young mice (Fig. 1E).

### Treatment with CCL24 Ab hindered Ang II–induced atrial arrhythmogenesis and electrical remodeling

**Fig. 2:**
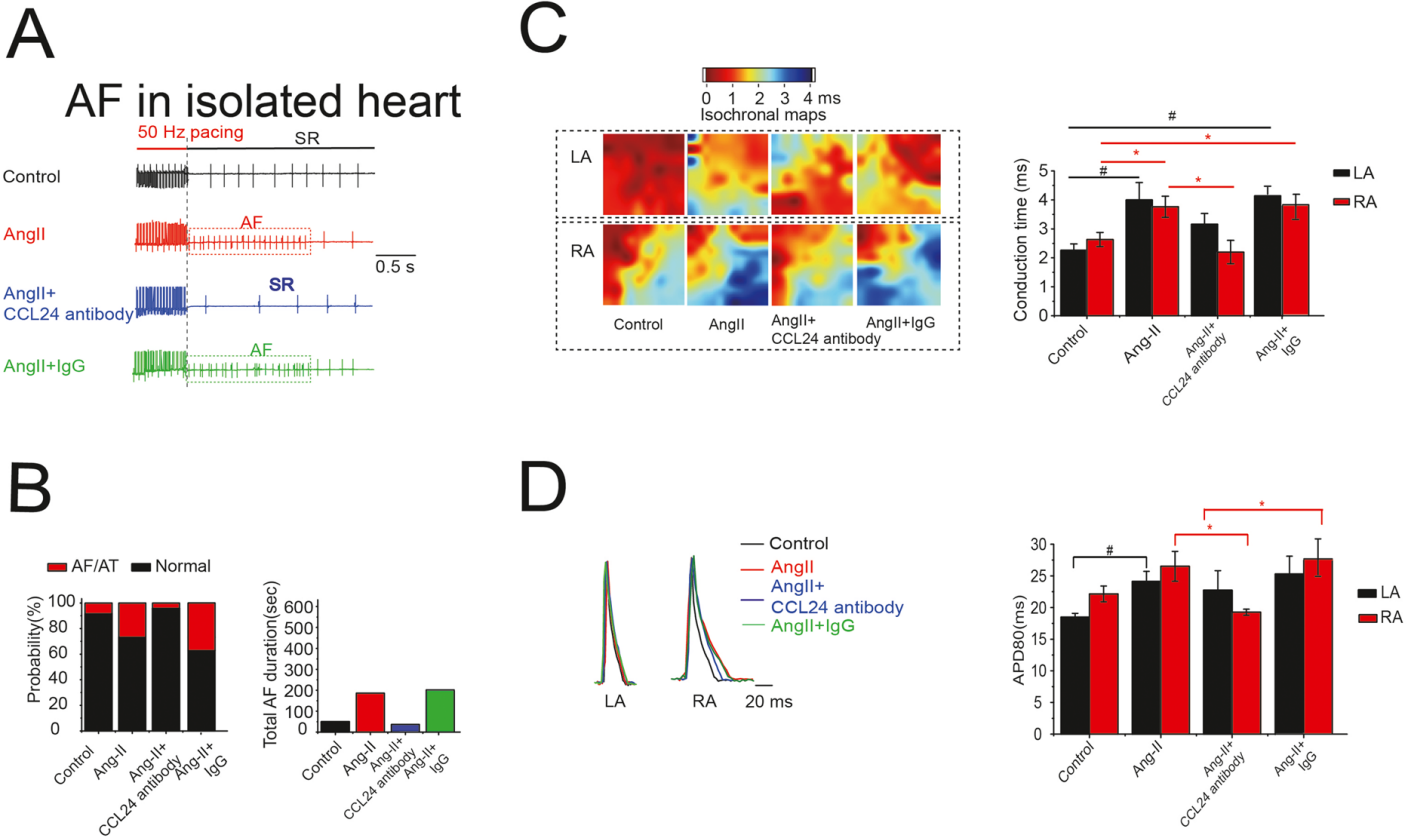
Treatment of CCL24 blocking antibody hindered angiotensin II–induced cardiac electrical remodeling. **A** Atrial electrogram recordings in response to burst pacing in Langendorff-perfused mice hearts. **B** Number of mice (of a total of 6 in each group) in which AF and/or atrial flutter could be reproducibly induced by right atrial (RA) burst pacing. (*n* = 6; *P* = 0.038 by Fisher’s exact test) and AF duration time induced by atrial burst pacing in mice. **C **High-resolution optical mapping to measure conduction velocity in Langendorff-perfused mice hearts. **D** The APD80 of atrial in Langendorff-perfused mice hearts

Langendorff-perfused mice hearts were subjected to in situ electrical and optical mapping. Administration of *CCL24* Ab prevents Ang II–induced atrial arrhythmia. Epicardium electrical stimulation induced atrial fibrillation/atrial flutter (AF/AFL) in 83.3% of Ang II–induced mice, but rarely in *CCL24* Ab mice. Total AF duration decreased from 173 s in Ang II–induced mice to 28 s (*p* < 0.05) in *CCL24* Ab mice (Fig. 2A–B). Ang II diminished the atrial conduction velocity in mice, and right atrial conduction velocity was increased in *CCL24* Ab mice (Fig. 2C). Action potential duration 80 (APD80) showed statistically significant increases in Ang II–induced mice atrium, and *CCL24* Ab recover the APD80 of mice right atrium (Fig. 2D).

### Treatment with CCL24 Ab hindered Ang II–induced cardiac hypertrophy and structural dysfunction

**Fig. 3:**
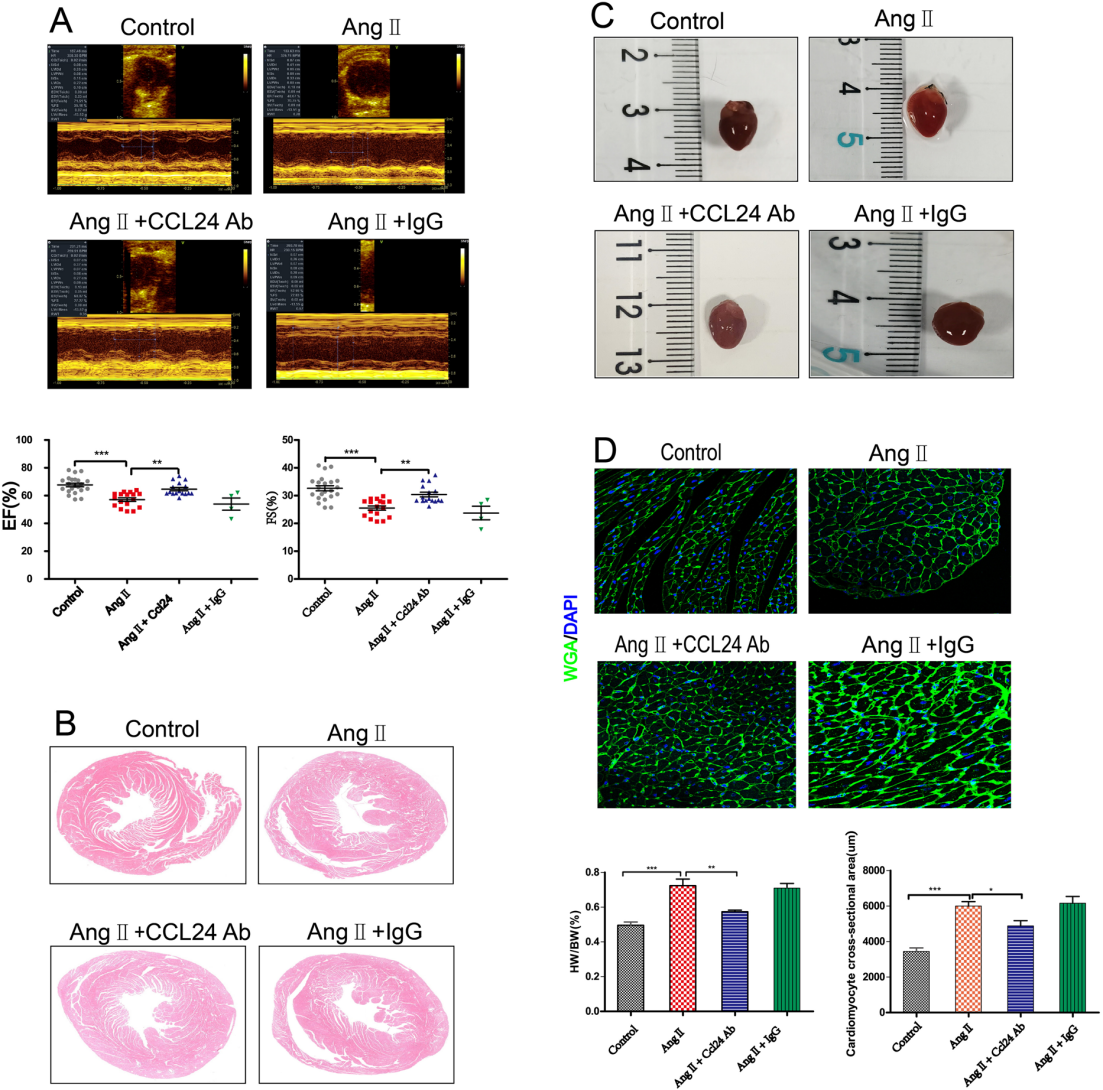
Treatment of CCL24 blocking antibody hindered angiotensin II–induced heart failure and cardiac hypertrophy. **A** M-mode echocardiography of left ventricular chamber and the measurement of ejection fraction (EF %) and fractional shortening (FS %). The data are represented as the means ± SEM (*n* = 16; one-way ANOVA; *P* < 0.05). **B** Hematoxylin and eosin staining of heart section. Scale bar in panel **A** is 1000 μm. **C** Representative heart size, and heart weight to body weight (HW/BW) ratio. The data are represented as the means ± SEM (*n* = 6; one-way ANOVA; *P* < 0.05). **D** TRITC-labelled wheat germ agglutinin staining of heart tissue sections, and quantification of cardiac-myocyte cross-sectional area (50 cells counted per heart). Scale bar in panel **C** is 50 μm. The data are represented as the means ± SEM (*n* = 6; one-way ANOVA; *P* < 0.0001)

Compared with Ang II–induced mice, *CCL24* Ab markedly prevented cardiac dysfunction induced by Ang II as reflected by the increasing left ventricular (LV) ejection fraction (EF) and fractional shortening (FS) in echocardiography (Fig. 3A). Moreover, as shown in Fig. 3B–C, *CCL24* Ab can mitigate cardiac hypertrophy during Ang II stimulation, which is indicated by the augment of heart size, heart weight to body weight (HW/BW) ratio, and the cross-sectional area of myocytes (Fig. 3D).

### Treatment with CCL24 Ab hindered Ang II–induced myocardial fibrosis

**Fig. 4:**
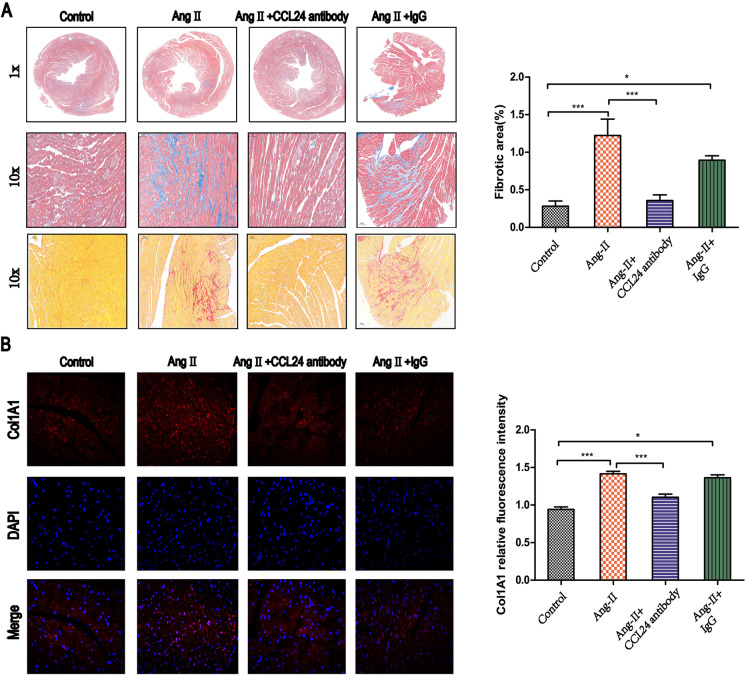
Treatment of CCL24 blocking antibody hindered angiotensin II–induced cardiac fibrosis. **A** Masson’s trichrome staining and Sirius red staining of myocardial fibrosis in heart tissues in mice. Scale bar in panel **A** lower part is 50 μm; scale bar in panel **A** upper part is 1000 μm; scale bar in panel **A** middle part is 200 μm. **B** Immunofluorescence of COL1A1 in heart tissue in mice. Scale bar in panel **B** is 20 μm. The data are represented as the means ± SEM (*n* = 6; one-way ANOVA; *P* < 0.05)

There was a significant decrease in cardiac fibrosis in *CCL24* Ab–treated animals compared with Ang II–induced mice according to both Masson stain and Sirius red stain in the same area of heart sections (Fig. 4A). Similarly, according to the immunofluorescence of *COL1A1* (Fig. 4B), we revealed that *CCL24* Ab significantly decreased the cardiac peripheral and interstitial collagen deposition during Ang II infusion. We revealed that *CCL24* plays a crucial role in Ang II–induced myocardial fibrosis and structural remodeling.

### RNA expression analysis and bioinformatics analysis of transcriptional profiling

**Fig. 5:**
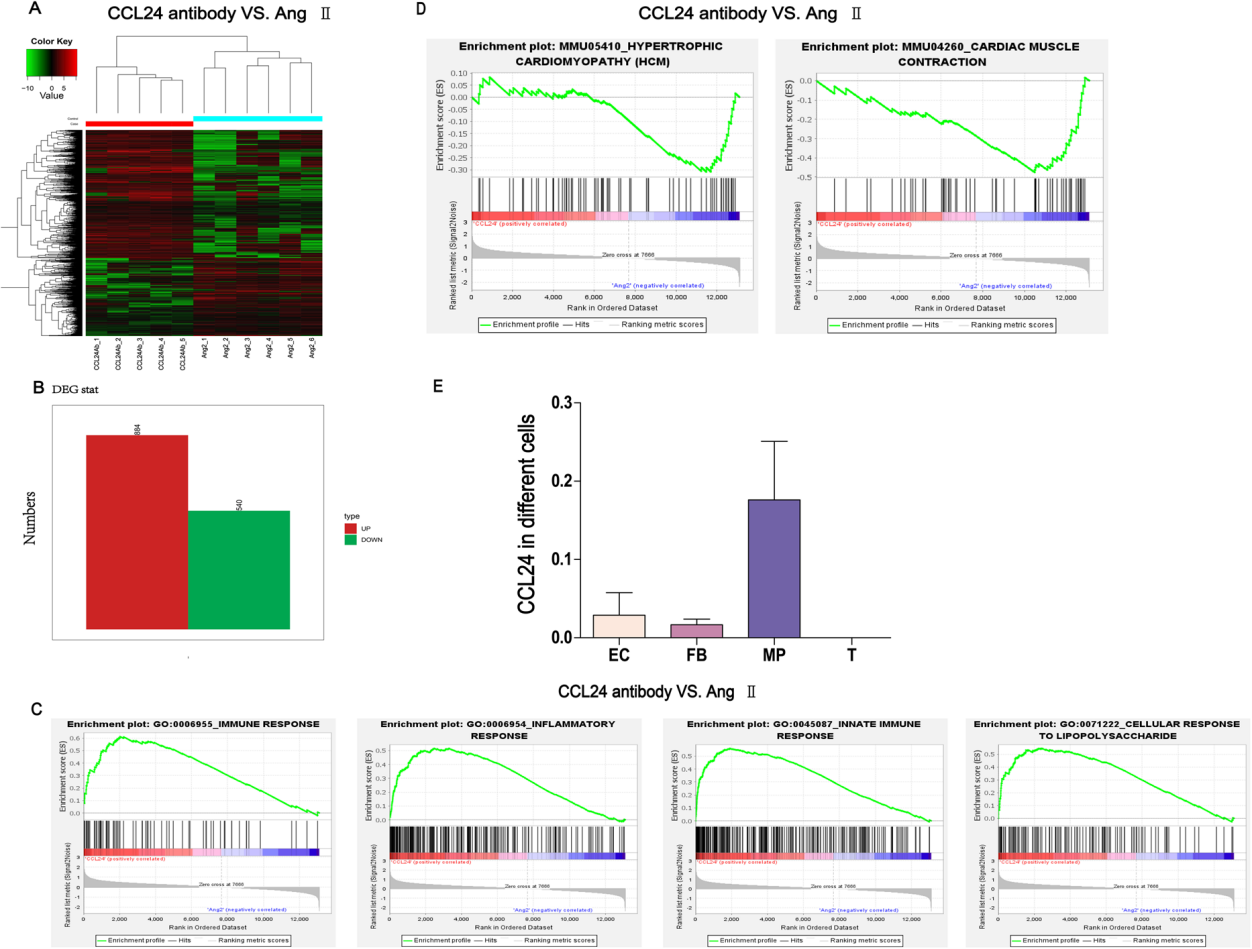
Data analysis of transcriptional profiling. **A** Heatmap of the differentially expressed genes. **B** The bar plot figure of the differentially expressed genes. **C** Gene set enrichment analysis (GSEA)–GO function enrichment plots of representative gene sets: *CCL24* Ab + Ang II group was related to immune response, inflammatory response, innate immune response, celluar response to LPS. **D** Gene set enrichment analysis (GSEA)–pathway enrichment plots of representative gene sets: *CCL24* Ab + Ang II group was negatively related to hypertrophic cardiomyopathy (HCM) and cardiac muscle contraction. **E** The mRNA expression of CCL24 in different cells

To investigate the mechanisms of gene programs activated in the cardiac tissue during the Ang II stimulation and *CCL24* Ab blocking, transcriptome analysis was performed ground on the RNA-seq data (Fig. 5A, B). As shown in Fig. 5C–D, GSEA (Gene Set Enrichment Analysis) found that the *CCL24* Ab group was related to immune response, inflammatory response, innate immune response, and cellular response to LPS, and the *CCL24* Ab group was negatively related to cardiac muscle contraction and hypertrophic cardiomyopathy (Fig. 5D). Figure 5E shows that the mRNA expression of CCL24 in the macrophage is higher than in the endothelial cell, fibroblast cell, and T cell.

### CytoF revealed the immune status in the mice’s heart

**Fig. 6:**
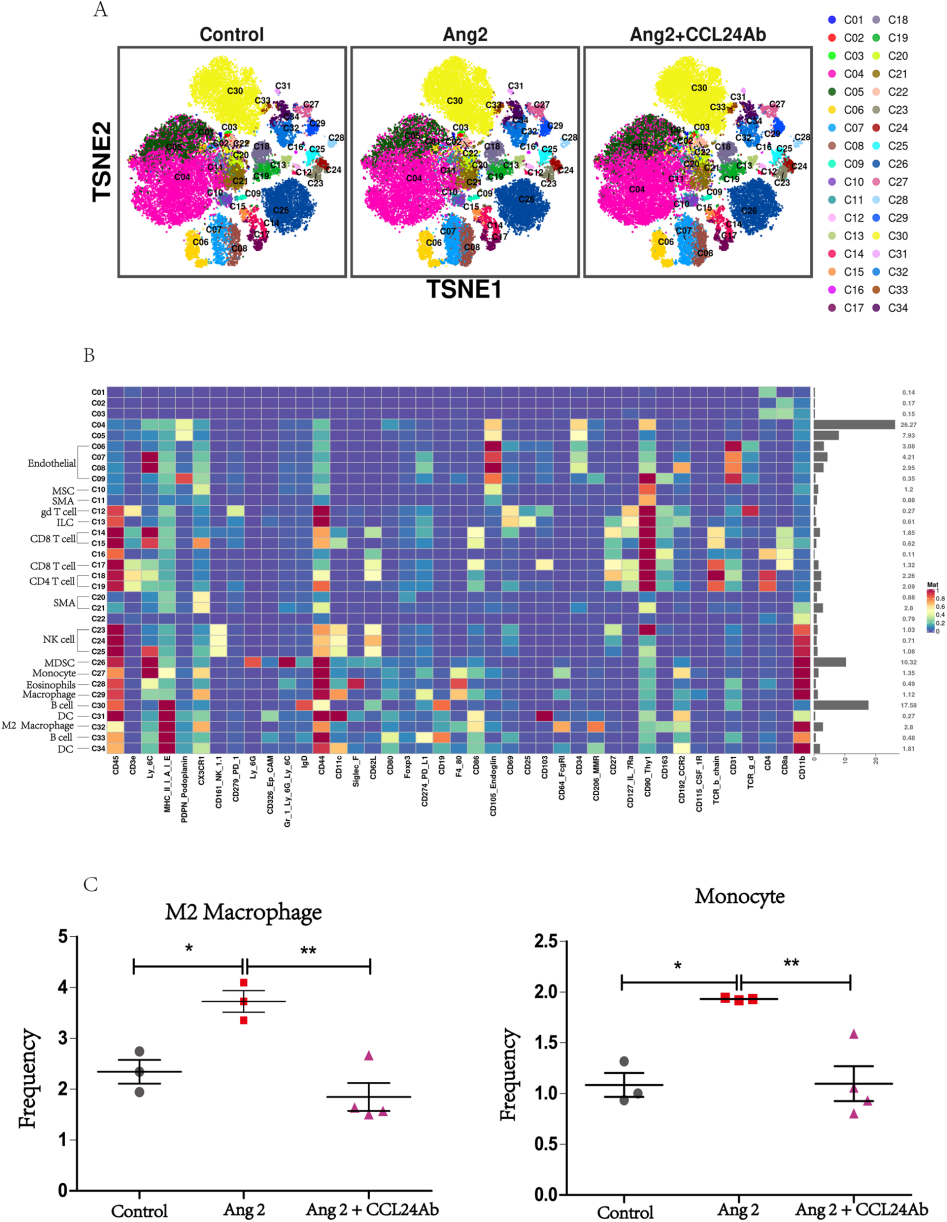
CytoF revealed the immune status in the mice heart. **A** The distribution and frequency of different immune cells in the heart. **B** The heatmap of different immune cells in the heart. **C** The proportion and frequency of M2 macrophages and monocytes in different groups. The data are represented as the means ± SEM (*n* = 3; one-way ANOVA; *P* < 0.05)

The basic immune status and difference in relative frequencies of cell types and molecular phenotypes in the mice heart were detected with CyTOF. Figure 6A–B reveal the distribution and frequency of different immune cells, including macrophage, monocyte, CD4^+^ T cell, CD8^+^ T cell, dendritic cell (DC), B cell, natural killer cell (NK), and eosinophil. Compared with the control group, the proportion and frequency of M2 macrophages and monocytes were significantly increased in mice in the Ang II group. While compared with the Ang II group, M2 macrophages and monocyte were significantly decreased in mice in the Ang II + CCL24 Ab group.

### Immunofluorescence co-localization of CCR3 receptor in different types of cells in myocardial tissue

**Fig. 7:**
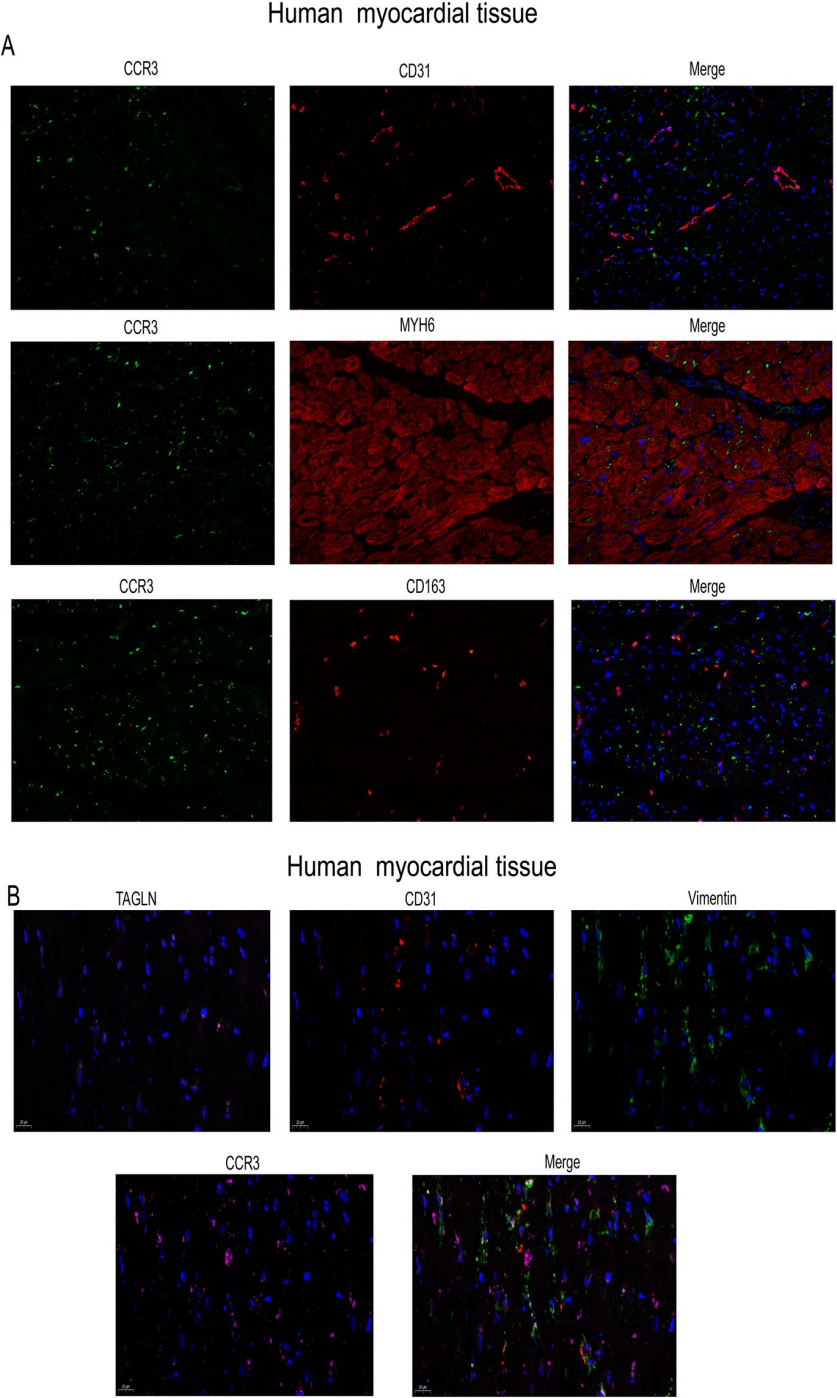
Immunofluorescence co-localization of CCR3 receptor. **A** Immunofluorescence co-localization of CCR3 receptor with endothelial cell, cardiomyocytes, and macrophage cell. **B** Immunofluorescence co-localization of CCR3 receptor with smooth muscle cell, endothelial cell, and fibroblast cell

As shown in Fig. 7A and B, we found that immunofluorescence co-localization of *CCR3* receptor is partly in cardiac fibroblast (vimentin) and macrophage cell (*CD163*), rather than in the endothelial cell (*CD31*), smooth muscle cell (*TAGLN*), and cardiomyocytes (*Myh6*).

### CCL24 promoted primary cardiac fibroblast activation

**Fig. 8:**
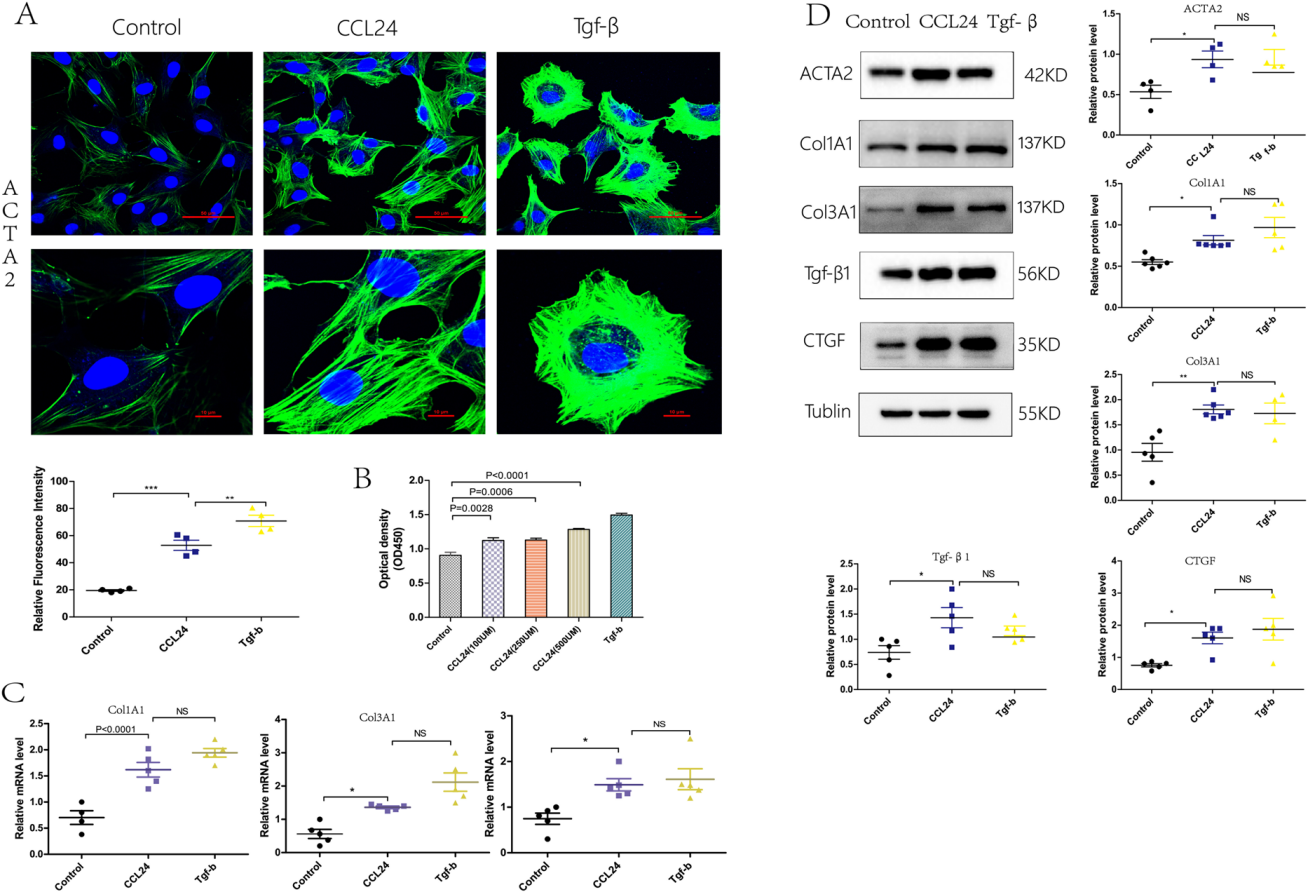
CCL24 promoted primary cardiac fibroblast activation. **A** Immunofluorescence of ACTA2 in heart tissues in mice. Scale bar in panel **A** upper part is 50 μm; scale bar in panel **A** lower part is 10 μm. **B** CCK8 counting assay of the proliferation of cardiac primary fibroblast. The data are represented as the means ± SEM (one-way ANOVA; *P* = 0.0028: control group vs. CCL24 100UM group, *P* = 0.0006: control group vs. CCL24 250UM group, *P* < 0.0001: control group vs. CCL24 500UM group). **C** Quantitative real-time polymerase chain reaction analysis of the mRNA levels of ACTA2, COL1A1 and COL3A1 in cardiac primary fibroblast with different stimulations. The data are represented as the means ± SEM (unpaired Student’s *t* test; *P* < 0.05: control group vs. CCL24 group). **D** Western blot analysis of ACTA2, COL1A1, COL3A1, Tgf-β, and CCN2 protein in in cardiac primary fibroblast under different stimulation. The data are represented as the means ± SEM (unpaired Student’s *t* test; *P* < 0.05: control group vs. CCL24 group)

At first, we extracted cardiac primary fibroblasts and identified them with an immunofluorescence marker (Supplementary Fig. 1A–B). Then, we performed assays to evaluate the activation and differentiation of cardiac fibroblast under stimulation of CCL24. As shown in Fig. 8A, *CCL24* promoted the fluorescence intensity of *α-SMA* in cardiac fibroblast, indicating that *CCL24* promotes fibroblast activation. In the meanwhile, the CCK8 counting assay reveals that *CCL24* could promote the proliferation of cardiac primary fibroblasts (Fig. 8B). Figure 8C shows that *CCL24* increased the mRNA expression of *ACTA2*,* CCN2(CTGF)*, *Col1a1*, and *Col3a1*. Then, we verified that *CCL24* promoted the differentiation of fibroblasts into myo-fibroblasts through enhancing the protein expression of *ACTA2*,* Col1A1*, *Col3A1*, *Tgf-β1*, and *CCN2(CTGF)* (Fig. 8D).

### CCL24 promoted the migration of primary cardiac fibroblast

**Fig. 9:**
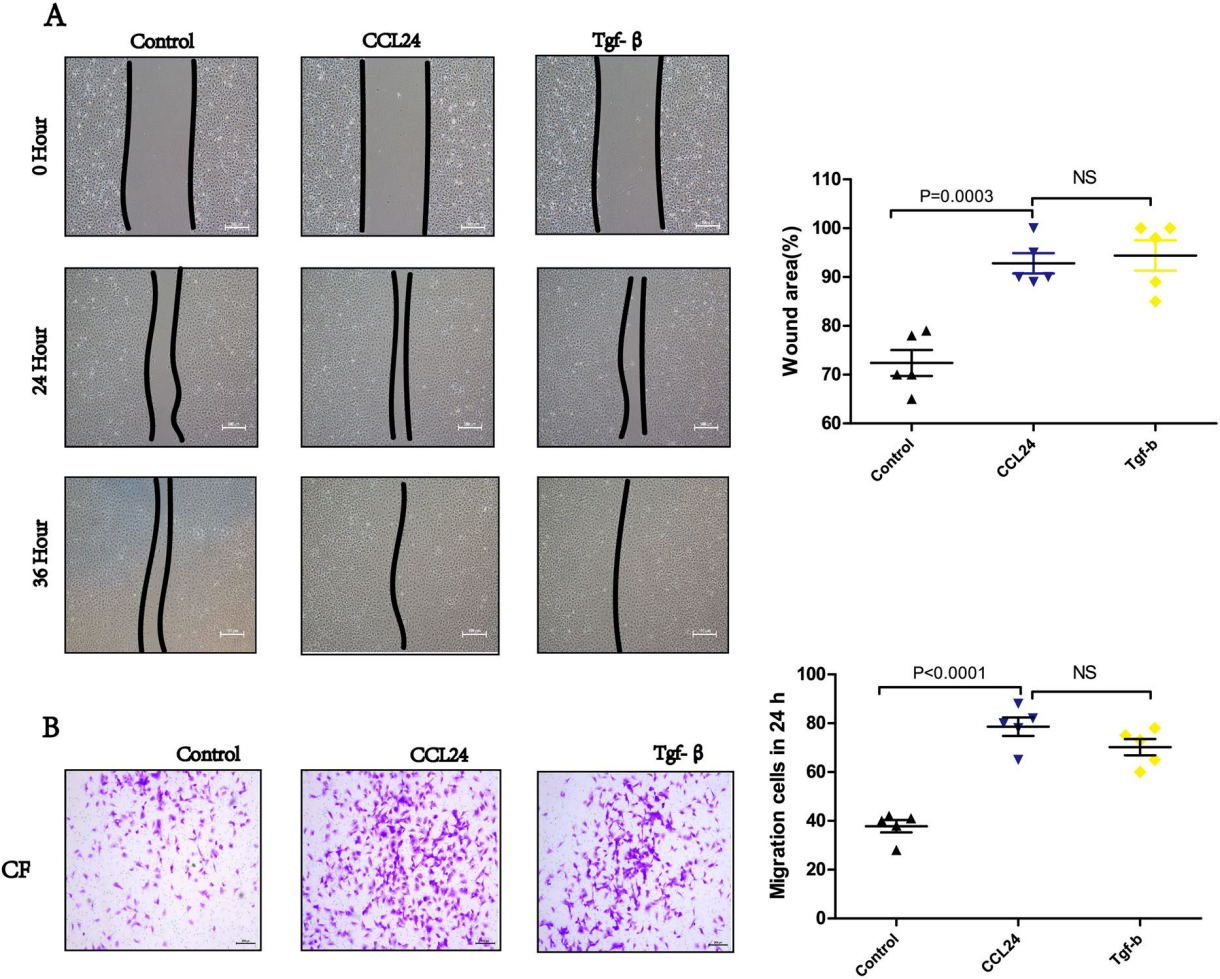
CCL24 promoted the migration of primary cardiac fibroblast. **A** Monolayer wound healing experiment of primary cardiac fibroblast under different stimulation. Scale bar in panel **A** is 100 μm. The data are represented as the means ± SEM (unpaired Student’s *t* test; *P* = 0.0003: control group vs. CCL24 100UM group). **B** Boyden chamber cell migration assay of primary cardiac fibroblast under different stimulation. Scale bar in panel **B** part is 200 μm. The data are represented as the means ± SEM (unpaired Student’s *t* test; *P* < 0.0001: control group vs. CCL24 100UM group)

We found that *CCL24* promotes the migration of primary cardiac fibroblast as revealed by scratch wound confluence (Fig. 9A). Subsequently, a transwell migration assay was performed to exclude the proliferation effect of *CCL24* in wound healing assay and further investigate its role in chemotactic migration. Transwell migration assay also revealed that *CCL24* significantly increased the ratio of migrating cells (Fig. 9B).

### RNA expression analysis of transcriptional profiling

**Fig. 10:**
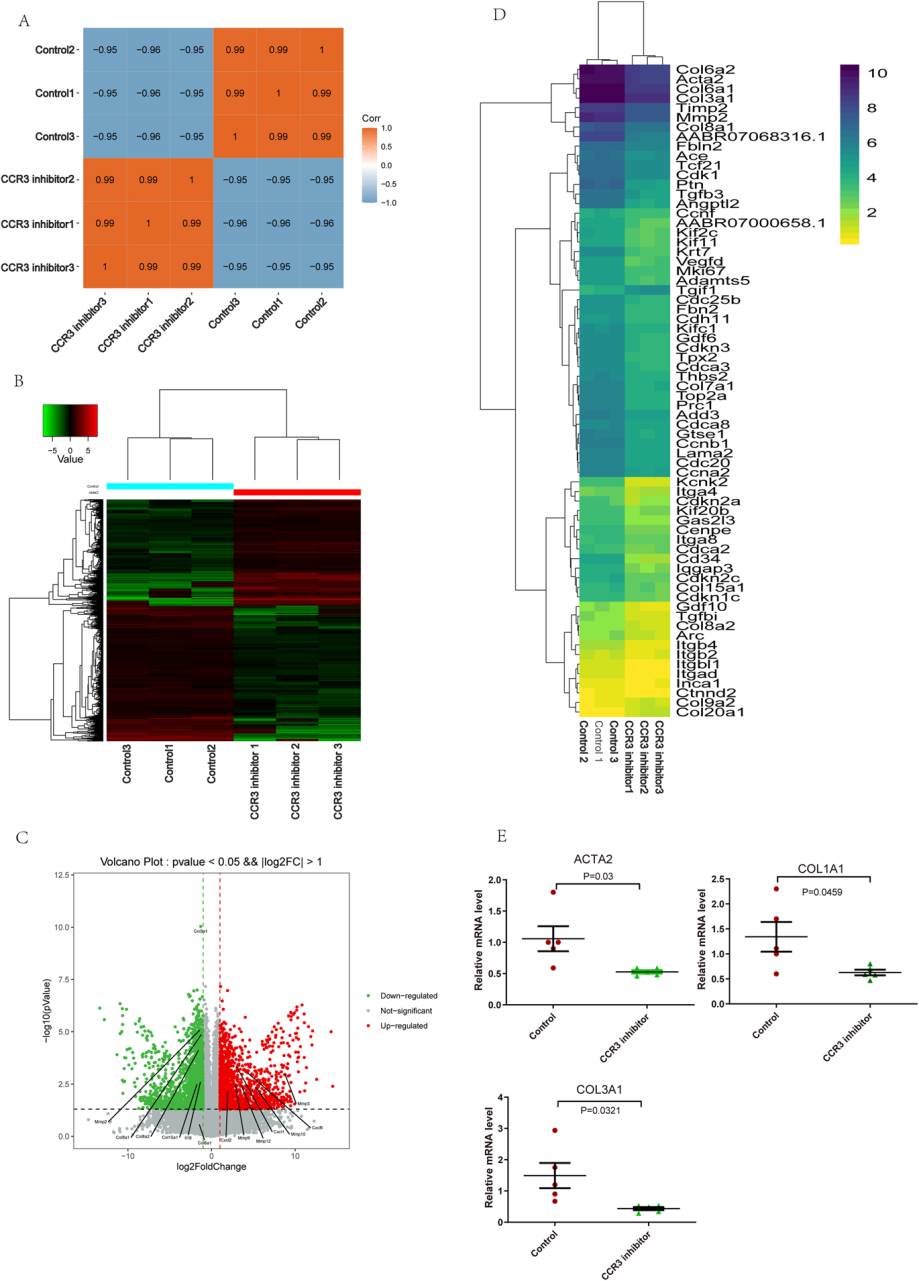
Data analysis of transcriptional profiling. **A, B, C** Transcriptome analysis was performed ground on the RNA-seq data. **D** Heatmap of the cardiac fibroblast activation related expressed genes. **E** Quantitative real-time polymerase chain reaction analysis of the mRNA levels of ACTA2, COL1A1, and COL3A1 in cardiac primary fibroblast under CCR3 inhibitor stimulation. These data are represented as the means ± SEM (unpaired Student’s *t* test; *P* < 0.05)

To investigate the gene programs activated in the cardiac fibroblast during the stimulation of *CCL24* and *CCR3* inhibitor, transcriptome analysis was performed ground on the RNA-seq data. As shown in Fig. 10A–D compared with the resting cardiac primary fibroblast, cardiac primary fibroblast decreased 996 genes during *CCR3* inhibitor stimulation which included *ACTA2*,* COL3a1*,* Tgf-β2*,* COL5a1*,* COL6a1*,* COL6a2*,* COL7a1*, and *COL8a1*. These genes are related with cardiac primary fibroblast activation. The transcriptome sequencing results of the CCR3 inhibitor were validated by quantitative RT-PCR (Fig. 10E).

### Bioinformatics analysis of differentially expressed genes

**Fig. 11:**
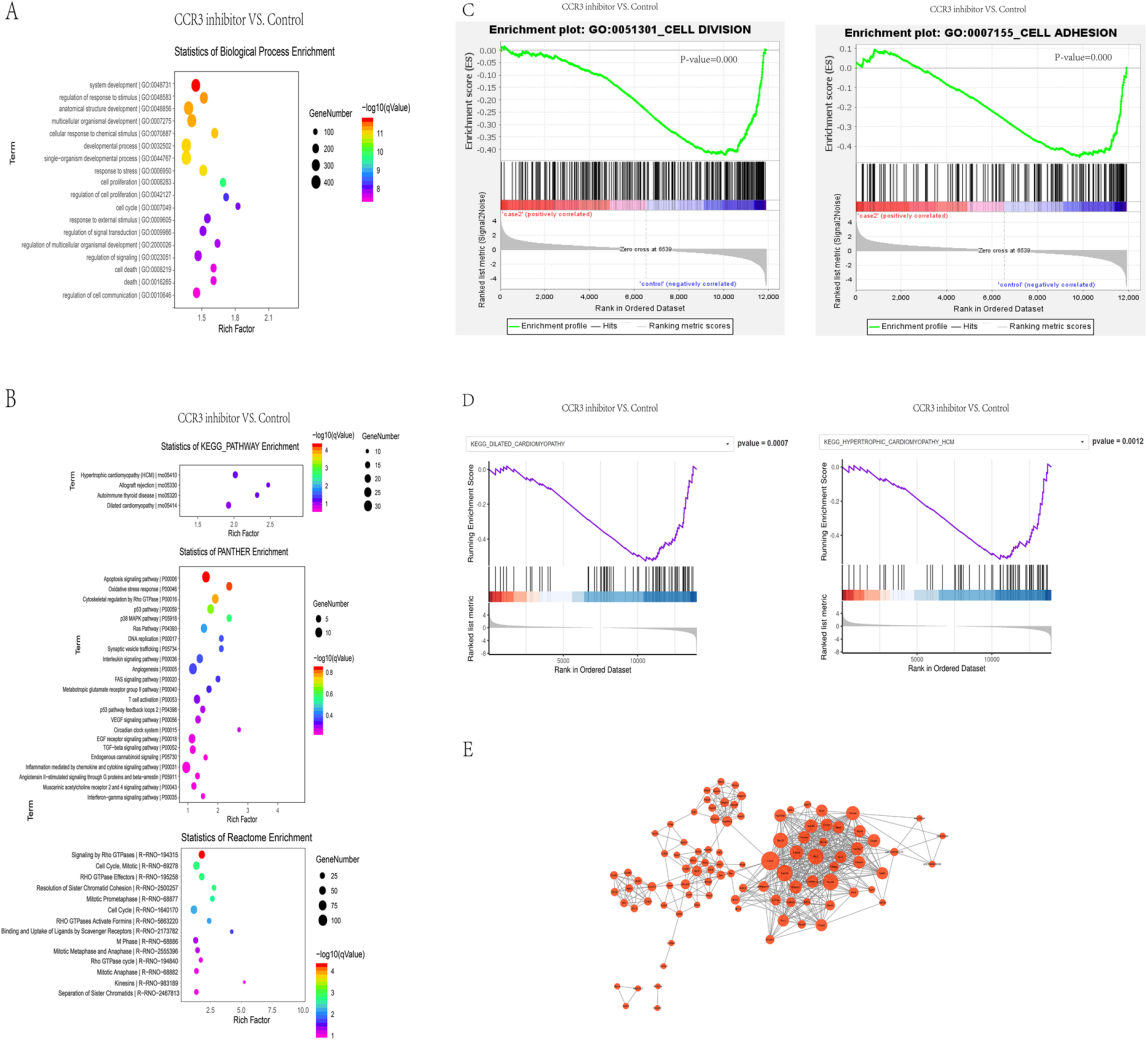
Bioinformatics analysis of differentially expressed genes. **A** The top 30 enrichment of the biological process results are listed. **B** The enriched signaling pathways from KEGG pathway, panther enrichment, reactome enrichment analysis. **C** Gene set enrichment analysis (GSEA)–GO function enrichment plots of representative gene sets from CCR3 inhibitor group and Control group: negative regulation of cell division, negative regulation of cell adhesion. **D** Gene set enrichment analysis (GSEA)–pathway enrichment plots of representative gene sets from CCR3 inhibitor group and control group: negative regulation of dilated cardiac cardiomyopathy (DCM) pathway, negative regulation of hypertrophic cardiomyopathy (HCM) pathway. **E** The PPI analysis of the differentially expressed genes

Bioinformatics analysis was performed of the differentially expressed genes in three groups on the basis of the Gene Ontology (GO) database. Figure 11A shows the top 20 enriched terms of differentially expressed genes in the developmental process, cell proliferation, cell cycle, cell death, etc. In terms of the signal pathway analysis of the enrichment database, and the top 30 are listed in Fig. 11B. “Cytoskeletal regulation by *Rho* *GTPase*,” “Signaling by *Rho* *GTPases*,” “*TGF-beta* signaling pathway,” and “*EGF* receptor signaling pathway” was obviously enriched, followed by signal transduction, chemokine signaling pathway, immune system, etc. Notably, “*TGF-beta* signaling pathway” and “*EGF* receptor signaling pathway” are closely related to malignant cardiac remodeling and myocardial fibrosis, and *Rho* *GTPases* signaling is closely associated with *CCL24/CCR3* axis. Then, we performed a GSEA analysis; we found that the *CCR3* inhibitor group was negatively related to cell division, cell adhesion, dilated cardiomyopathy, and hypertrophic cardiomyopathy (Fig. 11C–D). Therefore, *CCL24/CCR3* axis regulates cardiac primary fibroblasts activation and proliferation through its G protein–coupled receptor.

## Discussion

Cardiac injury causes cardiomyocyte necrosis and apoptosis in the heart, with concomitant activation of cardiac inflammation and reproduction of a reparative fibrotic scar that, for the near feature, guards against the rupture of the ventricular wall. Whereas, in long-term heart failure, myocardial interstitial fibrosis accumulates throughout the cardiac tissue, inducing cardiac wall and septal stiffening and gradually deteriorating cardiac structure and function (Tallquist and Molkentin 2017). Inflammation plays an important role in cardiovascular disease (CVD). According to the COLCOT Clinical Trials and LoDoCo2 Australian New Zealand Clinical Trials, compared with placebo, colchicine (0.5 mg/day) could reduce the risk of cardiovascular events prominently in patients with recent myocardial infarction or chronic coronary disease (Tardif et al. 2019; Nidorf et al. 2020). Accompanied by single-cell RNA-sequencing technology, we uncovered the heterogeneity of fibroblasts; especially, one cluster of fibroblasts from early muscle injury constituted the majority of *Cxcl5*^+^cluster cells and expressed inflammation-associated chemokine genes, for instance, *CCL2* and *CCL7* (Buechler et al. 2021). Therefore, in our study, we want to investigate the role of inflammation medium chemokine in the activation of cardiac fibroblast and cardiac fibrosis.

Chemokine occupies a vital position in the pathology of various CVD. Chemokine was initially discovered to attract several types of leukocytes which is the typical characteristic distinguishing chemokine from other cytokines (Zlotnik and Yoshie 2012). The *CXCL12/CXCR4* axis attaches great importance to myocardial repair after myocardial ischemia/reperfusion injury (Hu et al. 2007). Recently, the *CXCL1/CXCR2* axis has also been verified to participate in Ang II–induced cardiac dysfunction and myocardial remodeling (Wang et al. 2018). Numerous studies have contributed to our knowledge of chemokine in the cardiovascular system and CVD.

*CCL24* chemokine performs its biological functions by binding to its unique *CCR3* receptor, which was discovered to be a subgroup of class A G protein–coupled receptors (GPCRs) (Zlotnik and Yoshie 2012). *IL-4* in collaboration with *IL-10* synergistically induced macrophages and secreted plenty of *CCL24* (Makita et al. 2015) (Diny et al. 2016) which specifically induced M1 macrophage chemotaxis (Xuan et al. 2015), M2a macrophage activation, and tissue macrophage maintenance (Lee et al. 2020). *IL4/IL10* and *CCL24* were involved in an amplification loop in these inflammation cells. These inflammatory cells were shown to participate in cardiac remodeling (Frangogiannis 2019). In mouse and human hearts, regulatory T-cells (Treg) (Li et al. 2019) and macrophages (Wang et al. 2019) secreted a large amount of *CCL24* to promote the proliferation of cardiomyocytes in a paracrine manner. *CCL24* was previously verified to participate directly in the process of skin, pulmonary, and liver inflammation and fibrosis in humans (Segal-Salto et al. 2020; Mor et al. 2019). Overall, further studies are required to verify our hypothesis whether *CCL24* contributes to Ang II–induced cardiac dysfunction and myocardial remodeling.

We demonstrated firstly that *CCL24 /CCR3* axis is involved in cardiac remodeling during the pathology of heart failure. At first, we revealed that the expression of cardiac and plasma *CCL24* was higher in HF patients than in normal volunteers. And the cardiac electrical and structural remodeling have been partially alleviated with the *CCL24* monoclonal blocking antibody. The mechanisms of heart failure (HF) and atrial fibrillation (AF) are related to electrical and structural remodeling in the myocardium (atria and ventricular). Multiple re-entries due to shortening of the action potential are pivotal electrophysiological mechanisms of AF, the same heterogeneity of impulse conduction caused by atrial fibrosis. And similarly, the accumulation of myocardium fibrosis is considered to be the trait of structural remodeling in HF, and myocardium fibrosis is also the pathological basis for HF perpetuation. In our study, the *CCL24* blocking antibody could reduce the incidence and duration of atrial fibrillation; in mechanism, we found that the *CCL24-*neutralizing antibody recovers the APD80 of mice’s right atrium during Ang II stimulation. Inflammatory chemokine receptor *CCR3* (the unique receptor of *CCL24*) is one of the GPCRs (Dyer et al. 2019); the high-affinity agonists of CCR3 ligand, *CCL5/CCL11/CCL24/CCL26*, induced intracellular Ca^2+^ mobilization of cells. In optical mapping, the *CCL24* blocking antibody can stabilize its calcium current. In addition, we confirmed that Ang II diminished the atrial conduction velocity in mice, and right atrial conduction velocity was increased in *CCL24*-neutralizing antibody mice. This is one of the reasons of AF and in the meanwhile is one of the symbols of myocardium fibrosis in electrophysiological mapping. It is similar with our histological characteristics such as Masson staining and Sirius red stain. Furthermore, according to our bulk RNA-seq result, *CCL24/CCR3* axis was negatively related to muscle contraction, cardiac muscle contraction, heart contraction, and hypertrophic cardiomyopathy and immune and inflammatory response. These bioinformatics results are highly consistent with our molecular biology results; therefore, we can verify the participation of the *CCL24/CCR3* axis in the pathology of heart failure and cardiac fibrosis. Next, the single-cell analysis of cytometry by time of flight (CyTOF) revealed that CCL24 mAb decreased the M2 macrophage and monocyte polarization during Ang II stimulation. Then, immunofluorescence co-localization analysis confirmed the existence of CCR3 in macrophage and cardiac fibroblasts.

Thus, in vitro, we also proved that *CCL24/CCR3* axis is involved in the activation of cardiac primary fibroblast. Therapeutic targeting of *CCL24* hindered adverse cardiac reconstruction and pathology; therefore, providing an appealing novel approach for curing heart failure and cardiac fibrosis. However, in our study, we also found that the *CCL24* blocking antibody just alleviated partially the cardiac remodeling and dysfunction during the Ang II stimulation. In the future, we will explore the specific role of chemokine *CCL24* monoclonal antibodies in combination with other chemokine monoclonal antibodies in malignant cardiac remodeling.

### Conclusions

*CCL24/CCR3* axis plays a crucial role in the pathological development of cardiac remodeling during heart failure induced by Ang II. And inhibition of *CCL24* can be a potential therapeutic target in heart failure in mice of Ang II infusion. Protective effects of the *CCL24*-neutralizing antibody in the heart were associated with the reduction of M2 macrophage polarization and cardiac fibroblast activation within injured hearts.

### Limitations

There have several limitations in our study. First of all, we just only verify the role of the *CCL24/CCR3* axis in heart failure with monoclonal blocking antibody; however, we do not verify it with transgenic mice. Furthermore, the direct function of the *CCR3* receptor in heart failure has not been clarified; in the future, we need more research on the *CCR3* receptor in the cardiovascular system.

## Methods and related materials

### Heart failure patients and control subjects

Nine patients (5 men and 4 women) with chronic symptomatic heart failure were studied. Control subjects were seven healthy donors (4 men and 3 women). We cut out parts of the myocardium of heart failure patients when they received heart transplants. In addition, we obtained the control myocardium with the donor’s heart. We described the clinical baseline data of patients in Table 1.

**Table 1:** Clinical baseline data of the patients involved in this study

	Sex	Age	Heart disease	EF (%)	LAD (mm)
HF					
	M	42	CHD	45	40
	M	71	MR\AR	40	53
	M	65	MR\AR	40	40
	M	66	MS\MR	56	50
	M	39	MR\AR	56	61
	F	63	AS MR AR	59	46
	F	70	MR\AR	43	40
	F	39	MS\MR	50	43
	F	58	MR\AR	42	40
Non-HF					
	M	50	AD	61	35
	M	47	MR MS AR	61	45
	M	64	AS AR	68	37
	M	38	MR	54	27
	F	75	TR	77	57
	F	69	MS MR AS AR	59	29
	F	36	MR	57	33

*HF* heart failure, *HD* heart disease, *EF* ejection fraction, *LAD* left atrial dimension, *CHD* coronary heart disease, *MR* mitral regurgitation, *MS* mitral stenosis, *AR* aortic regurgitation, *AS* aortic stenosis, *AD* aortic dissection, *TR* tricuspid regurgitation

### Mice models

Six to eight weeks of age C57BL/6 J wild-type mice (male) were used in our study. We injected Ang II (1500 ng/kg/min) for 15 days subcutaneously to establish a heart failure model. The *CCL24* blocking antibody was administrated intravenous injection to mice beginning 1 day after Ang II injection and continued three times a week during Ang II stimulation**.** We killed all the mice by cervical dislocation after isoflurane anesthesia (5 vol%).

### Reagents and antibodies

*CCL24* and *TGF-β* were acquired in Sigma Chemical Co. Primary antibodies against *CCR3*,* CCL24*,* Col1a1*,* Col3a1*,* CCN2*,* a-SMA*,* TGF-β*, and *CCN2(CTGF)* were collected from Cell Signaling Technology (CST). We obtained α-tubulin antibodies from Abcam. We purchased goat IgG-HRP antibodies (anti-rabbit and anti-mouse) from CST. The blocking antibody of *CCL24* was purchased from PEPROTECH.

### Echocardiographic

We performed an echocardiographic assay using a high-frequency linear array transducer, and we obtain two-dimensional images at mid-papillary and apical levels, and an echocardiographic assay was performed for anesthetized mice. We obtained end-diastolic and end-systolic left ventricular volumes by biplane area–length method. Then, the left ventricular ejection fraction was calculated automatically.

### Langendorff-perfused isolated mice hearts

We killed mice humanely through intraperitoneal injection with pentobarbital sodium. The heart was mounted rapidly onto a Langendorff apparatus. Then, perfused the isolated hearts with a Tyrode solution at the flow rate of 3 mL/min at 37 °C.

### Electrical mapping of isolated heart

We recorded extracellular potentials (ECP) from epicardium in a multi-electrical array (MEA) mapping system (Mapping Lab Ltd., UK). Left and right atrial simultaneous recordings were performed with two 64-channel MEA. To assess atrial fibrillation (AF) inducibility, the stimulator was placed on the left atrium with burst pacing. The detail of the burst pacing is as follows: 10 s burst pacing at a cycle length of 20 ms with a pulse duration of 5 ms, and then stabilized for 4 min. Repeated burst pacing 8 times with 4 min of stabilization each time. AF was defined as a rapid and irregular atrial rhythm (rapid and irregular ECP) lasting at least 1 s.

### High-resolution optical mapping of isolated hearts

We performed high-resolution optical mapping of isolated mice hearts. In brief, to arrest cardiac motion using excitation–contraction uncoupler Blebbistatin (10 μM). Then, load the membrane potential fluorescent dye Rh237 and calcium fluorescent dye Rhod2-AM to optically map membrane potential and calcium signal. We captured fluorescence changes with a high-speed camera (900 frames/s, OMS-PCIE-2002, Mapping Lab Ltd.), and the spatial resolution in our experiments was 128-by-128 pixels. We analyzed related data with O Map Scope 5.0 software (Mapping Lab Ltd.).

### Immunohistochemistry assay

We fixed the heart tissues with 4% paraformaldehyde and then embedded the heart tissues in paraffin. An immunohistochemistry assay was carried out with paraffin sections to evaluate hypertrophy, fibrosis, and inflammation of the heart. We dehydrated heart sections in the graded methanol series. Then antigen retrieval was performed using a microwave in citrate buffer. Then, we washed the sections in PBS, and we incubated the sections with 3,3-diaminobenzidine tetrahydrochloride (DAB) for several minutes.

### Real-time quantitative polymerase chain reaction (RT-qPCR)

We extracted RNA with TRIZOL as described by the manufacturers’ instructions. We reverse-transcribed 500 ng of RNA with SuperScript VILO DNA Synthesis Kit (Takara). We carried out real-time (RT) qPCR with an ABI RT PCR Detection System with SYBR Green Supermix (Takara). We analyzed the expression of target genes using the ΔΔCt method.

### Western blotting

We subjected proteins by sodium dodecyl sulfate–polyacrylamide gels. Then, we transferred the proteins to polyvinylidene difluoride membranes. Followed by blocking with 5% non-fat milk for 60 min, these membranes were incubated with the primary antibodies for 4 h at room temperature (RT). Next, membranes were incubated with secondary antibodies for 60 min at RT. The membranes were visualized by electro-chemiluminescence plus reagent (Merck Millipore Ltd.). The intensity was quantified with Chemiluminescence Imaging System (SHST).

### Isolation and cell culture of cardiac primary fibroblasts

We isolated cardiac primary fibroblasts with the cold trypsin digestion method according to our protocol and grew with Fibroblast Medium-2 (FM-2; ScienCell).

In brief, myocardial tissues from neonatal SD rats were incubated with the 4 °C trypsin for 10 h; then, these myocardial tissues were digested with the warm trypsin every 5 min which was performed five times repeatedly. Then, cardiac primary fibroblasts were collected with density gradient centrifuging.

### Monolayer wound healing experiments

Cardiac primary fibroblasts in 6-well plates grew to 80–90% confluence and then were serum-starved for 24 h. Then, one scratch in each well was made using a 100–1000 μl tip. After being rinsed twice with phosphate buffer saline, we incubated cardiac primary fibroblasts with FM-2. Then, we took images of the same regions at 0 or 24 h after stimulation and analyzed them.

### Cell migration assay with Boyden transwell chamber

We performed migration assay with Boyden transwell chamber (8 µm pore size membrane filter, Corning, NY, USA). Cardiac primary fibroblasts (2 × 10^4^) were grown in the upper chamber. The medium used in the upper chamber is FM-2 containing 0.5% FBS. The medium used in the lower chamber is FM-2 containing 10% FBS. After incubation, we fixed these fibroblasts which migrated to the bottom surface of the filter; then, the membranes on slides were mounted with a mounting medium. Finally, we quantified and analyzed the number of migrating fibroblasts from microscopic images of transwell membranes.

### Immunofluorescence staining

We fixed cells with 4% paraformaldehyde for 15 min at room temperature, and rinsed cells twice with PBS. Then, to permeabilize cell membranes, we treated cells with 0.1% Triton X-100 for 5 min. 5% BSA was added to cells and let stand 1 h at room temperature to block nonspecific sites of antibody adsorption. Cells were then incubated with the primary antibody overnight at 4 °C, then rinsed cells three times with PBS. Then, cells were incubated with fluorophore-conjugated secondary antibody for 1 h in the aluminum foil at RT. We washed cells twice with PBS; then, nuclei were stained with DAPI for 10 min. All the samples were rinsed with PBS again and finally observed using a confocal microscope.

### RNA-seq

We extracted total RNA from cardiac tissues and cells with RNeasy mini Kit (Qiagen), and then we prepared RNA samples using TruSeq RNA Sample Preparation Kit to construct mRNA library and deep sequencing according to related protocol. Finally, we performed cluster formation and sequencing on the HiSeq X Ten platform on the basis of standard sequencing protocols.

### Bioinformatic analysis of RNA-seq data

Bioinformatics analysis was performed of the differentially expressed genes in three groups on the basis of the GSEA (Gene Set Enrichment Analysis) and Gene Ontology (GO) database. The functional assignments were mapped onto Gene Ontology (GO). Genes were compared with the Kyoto Encyclopedia of Genes and Genomes database (KEGG) by using BLASTX4 at *E* values <  = 1e − 10. Then, a Perl script program was used to retrieve KO information from the blast results, and associations between genes and pathways were established. KEGG pathway and GO enrichments were analyzed in a command-line program KOBAS 2.0. We used the whole genome as the default background distribution to identify the significantly enriched pathways statistically in a set of sequences. For each pathway that occurs in the set of genes, we counted the total number of genes in the set that were involved in the pathway term or the GO term. We calculated the *p* value using a hypergeometric distribution.

### CytoF

Take out the mouse heart tissue from the tissue storage solution and wash twice using a cell 1640 culture medium. Cut the tissue into 1 mm^3 pieces. Collect the pieces into a centrifuge tube and add digestive enzyme mix (C-IV (2 mg/mL) + H (250 µg/mL) + D (20 µg/mL), and fill with cell culture medium up to 5 mL. Incubate the tissue soup in a shaking incubator at 37℃ for 1 h. Filter the dissociated tissue through the 70 μm cell strainer. Centrifuge these cells at 400 g for 5 min at 4℃ to collect the cells. Aspirate the supernatant and resuspend the cells in FACS Buffer and count cell number. Using 36% Percoll to remove debris by centrifuging at speed of 400 g 30 min. Add 103Rh + mouse filler splenocytes (2021-Nature Protocols-Mass cytometry profiling of human dendritic cells in blood and tissues) into mouse heart single-cell suspensions. Prepare Cell-ID Cisplatin Solution in PBS (final concentration of 250 nM 194Pt), resuspend cells in 100 μL Cisplatin Solution, and incubate for 5 min on ice. Wash cells twice by adding 1 mL FACS Buffer to each tube, centrifuge at 400 g for 5 min at 4 ℃, and discard supernatant by aspiration. Add Fc-receptor blocking solution to each tube and incubate for 20 min on ice. Prepare Antibody Cocktail in FACS Buffer (see Antibody Cocktail Table), add 50 μL of the antibody cocktail to each tube, and incubate for 30 min on ice. Following the incubation, wash cells twice by adding 1 mL FACS Buffer to each tube, centrifuge at 400 g for 5 min at 4 ℃, and discard supernatant by aspiration. Prepare cell intercalation solution in Maxpar Fix and Perm Buffer (final concentration of 250 nM 191/193Ir. Add 200 μL of the cell intercalation solution to each tube and gently vortex, leave overnight at 4 ℃. Wash cells by adding 1 ml FACS Buffer, centrifuge at 800 g for 5 min at 4 ℃, and discard supernatant by aspiration. Wash cells with 2 mL of deionized water, centrifuge at 800 g for 5 min at 4 ℃, and discard supernatant by aspiration. Resuspend cells in 1–2 mL deionized water, count cells. Centrifuge at 800 g for 5 min at 4 ℃ and discard supernatant by aspiration. Samples are ready for CyTOF. Turn on the CyTOF, ensemble sample introduction system. Run a Tuning and QC procedures to calibrate the CyTOF using Tuning solution and EQ beads. Resuspend and adjust cell concentration to 1 × 10^6/mL with deionized water containing 20% EQ beads. Transfer cells through a 40-μm filter to a new FACS tube, acquire data on a CyTOF system. Data of each sample were debarcoded from raw data using a doublet-filtering scheme with unique mass-tagged barcodes. Each.fcs file generated from different batches was normalized through bead normalization method. Manually gate data using FlowJo software to exclude to 103Rh + splenocytes, debris, dead cells, and doublets, leaving live single cells. Apply the X-shift clustering algorithm to all cells to partition the cells into distinct phenotypes based on marker expression levels. Adjust clustering parameters to obtain suitable number of clusters. Annotate cell type of each cluster according to its marker expression pattern on a heatmap of cluster vs marker. Use the dimensionality reduction algorithm t-SNE to visualize the high-dimensional data in two dimensions and show distribution of each cluster and marker expression and difference among each group or different sample type. Perform T-test statistical analysis on the frequency of annotated cell population.

### Statistical approach

Experimental data were displayed as mean ± SD and *p* ≤ 0.05 means statistically significant. Means of two groups were compared using Student’s *t* test (unpaired, 2-tailed), and for comparison of more than two groups, one-way ANOVA was used. We performed statistical analysis with SPSS 20.0 statistical software.

### Ethical statement

All mice experiments were performed according to the guidelines from Directive 2010/63/EU of the European Parliament on the protection of animals used for scientific purposes, and all animal procedures were approved by the Animal Care and Use Committee of Zhejiang University. Informed consent was obtained from all participants in accordance with the guidelines of the Human Subjects Committee of the Medical Ethical Commission of the First Affiliated Hospital of Zhejiang University (China) and the Declaration of Helsinki.

## Supplementary Information

Below is the link to the electronic supplementary material.

Supplementary file1 (JPG 1107 KB)

Supplementary file2 (JPG 22047 KB)

## Data Availability

All data generated and/or analyzed during this study are included in this published article.

## Abbreviations

Ang II: Angiotensin II
*CCL24*: Chemokine c–c motif ligand 24
*CCR3*: C-C chemokine receptor type 3
ECM: Extracellular matrix
WT: Wild type
CHF: Congestive heart failure
EDV: End-diastolic volumes
ESV: End-systolic volumes
MEA: Multi-electrical array
DAB: Diaminobenzidine tetrahydrochloride
HW/BW: Heart weight/body weight
ANF: Atrial natriuretic factor
BNP: Brain natriuretic peptide
EF: Ejection Fraction
FS: Fractional shortening
LV: Left ventricular
CVD: Cardiovascular diseases
GTP: Guanosine triphosphate
GPCR: G protein–coupled receptor
Treg: Regulatory T-cells
*SMA*: Smooth muscle actin
*CCN2*: Connective tissue growth factor
