# Supplementary figures and images for: CCL24/CCR3 axis plays a central role in angiotensin II–induced heart failure by stimulating M2 macrophage polarization and fibroblast activation
Source: Cell Biol Toxicol. 2022 Sep 22;39(4):1413–31. doi: 10.1007/s10565-022-09767-5 (PMC10425496; doi:10.1007/s10565-022-09767-5)

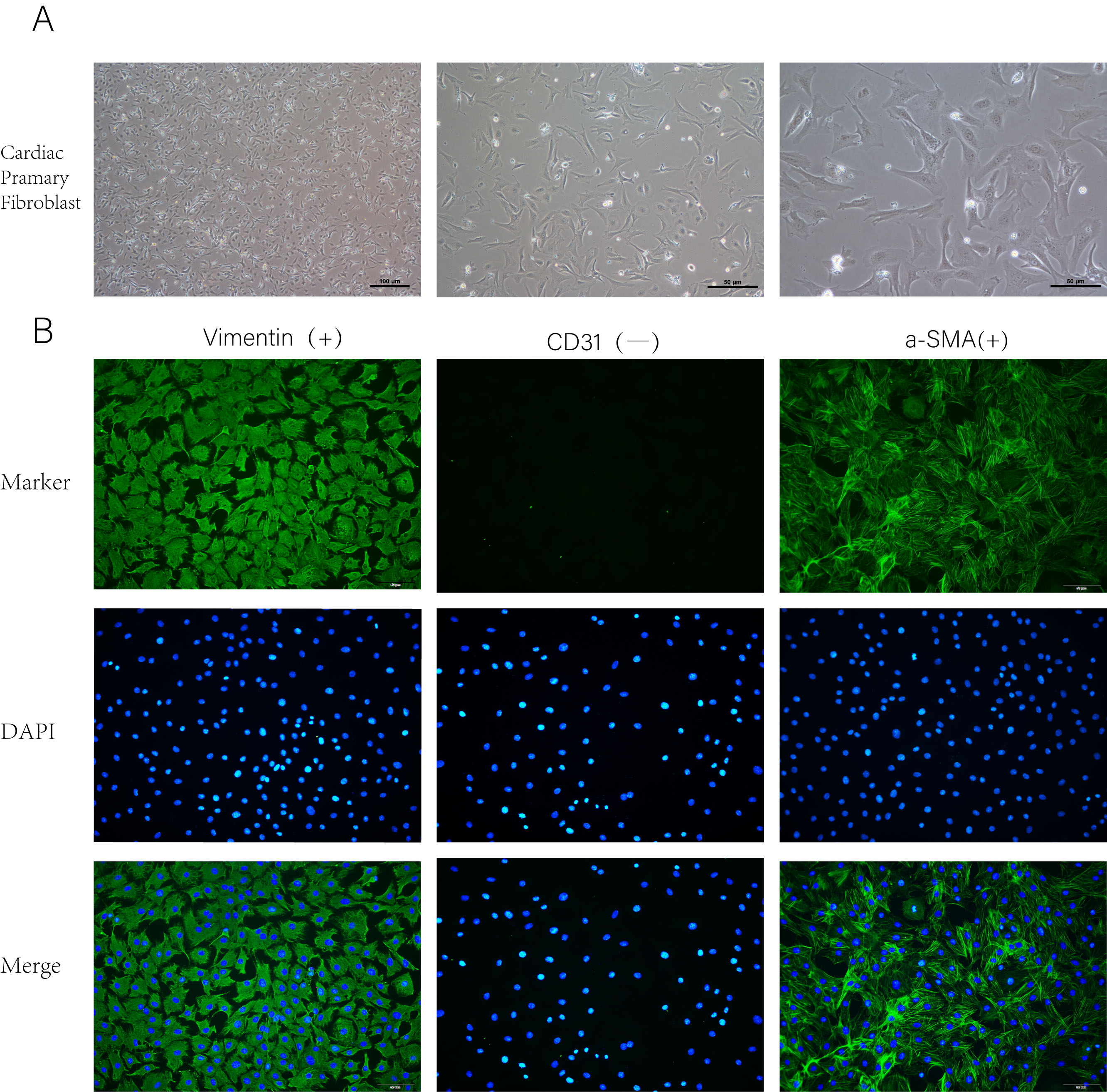

Supplement: Supplementary file 1 — Supplementary file1 (JPG 1107 KB) [file 10565_2022_9767_MOESM1_ESM.jpg]
